# Identification of Baicalin as an Immunoregulatory Compound by Controlling T_H_17 Cell Differentiation

**DOI:** 10.1371/journal.pone.0017164

**Published:** 2011-02-16

**Authors:** Ji Yang, Xue Yang, Yiwei Chu, Ming Li

**Affiliations:** 1 Department of Dermatology, Zhongshan Hospital, Fudan University, Shanghai, China; 2 Division of Rheumatology, Huashan Hospital, Fudan University, Shanghai, China; 3 Department of Immunology, Shanghai Medical College, Fudan University, Shanghai, China; Institut Jacques Monod, France

## Abstract

T_H_17 cells have been implicated in a growing list of inflammatory disorders. Antagonism of T_H_17 cells can be used for the treatment of inflammatory injury. Currently, very little is known about the natural compound controlling the differentiation of T_H_17 cells. Here, we showed that Baicalin, a compound isolated from a Chinese herb, inhibited T_H_17 cell differentiation both in vitro and in vivo. Baicalin might inhibit newly generated T_H_17 cells via reducing RORγt expression, and together with up-regulating Foxp3 expression to suppress RORγt-mediated IL-17 expression in established T_H_17 cells. In vivo treatment with Baicalin could inhibit T_H_17 cell differentiation, restrain T_H_17 cells infiltration into kidney, and protect MRL/lpr mice against nephritis. Our findings not only demonstrate that Baicalin could control T_H_17 cell differentiation but also suggest that Baicalin might be a promising therapeutic agent for the treatment of T_H_17 cells-mediated inflammatory diseases.

## Introduction

The T helper 17 (T_H_17) lineage, a lineage of effector CD4^+^ T cells characterized by production of interleukin (IL)-17, is described based on developmental and functional features distinct from classical T_H_1 and T_H_2 lineages [1], [2]. T_H_17 cells are associated with the development and pathogenesis of a growing list of chronic inflammatory diseases, including rheumatic arthritis, psoriasis, atopic dermatitis, and asthma [3], [4], [5]. Our studies, as well as others, have shown that T_H_17 cells also play a key role in the pathogenesis of systemic lupus erythematosus (SLE) [6], [7], [8], [9], [10], [11]. Several studies have advocated that T_H_17 cells might be a promising therapeutic target for chronic inflammatory injury [12], [13].

The differentiation of T_H_17 cells is initiated by transforming growth factor-β (TGF-β) and interleukin-6 (IL-6) in mice, and interleukin-23 (IL-23) is also required [14]. Signal transduction and activator of transcription 3 (STAT3), aryl hydrocarbon receptor (AHR) and the retinoic acid-receptor-related orphan receptor-γt (RORγt) mediate T_H_17 lineage commitment [15], [16], [17]. Several studies have indicated that 2,3,7,8-tetrachlorodibenzo-p-dioxin (TCDD), halofuginone, and retinoic acid could suppress the expression of these transcription factors, and subsequently inhibit the differentiation of T_H_17 cells [18], [19], [20]. However, few natural compounds restraining T_H_17 cells are known. Moreover, it is important to explore not only effective but also safe therapeutic agents for the treatment of T_H_17 cells-mediated inflammatory injuries.

Baicalin, which is a main active ingredient originally isolated from the root of Huangqin (*Scutellaria* baicalensis Georgi), has safety records in clinic and has been used as an anti-inflammatory drug in traditional Chinese medicine [21], [22]. Previous studies have showed that Baicalin could inhibit the proliferation of mononuclear cells, inhibit macrophage activation, inhibit the production of T_H_1 related cytokines in different disease murine models [23], [24]. Baicalin was shown to reduce the severity of experimental autoimmune encephalomyelitis (EAE) [23]. Since T_H_17 cells are important inducer of EAE, we hypothesized that Baicalin might inhibit inflammatory injuries by suppressing effector T_H_17 cells. Furthermore, previously published data confirmed that Baicalin inhibited the activation of AHR [25], which might has relevance to the proposed effect on T_H_17 cell development.

In this study, we observed that Baicalin inhibited T_H_17 cell differentiation in vitro. Detailed studies showed that Baicalin might inhibit newly generated T_H_17 cells via suppressing RORγt expression, and together with up-regulating Foxp3 expression to suppress RORγt-mediated IL-17 expression in established T_H_17 cells. Baicalin could inhibit the generation of T_H_17 cells in vivo, reduce T_H_17 cells infiltration into kidney via inhibition of the CCL20-CCR6 signaling pathway, and could protect lupus-prone MRL/lpr mice against nephritis. Taken together, these findings suggest that Baicalin might be a promising therapeutic agent for the treatment of T_H_17 cells-mediated inflammatory diseases.

## Results

### Baicalin inhibits T_H_17 cell differentiation in vitro

**Figure 1:**
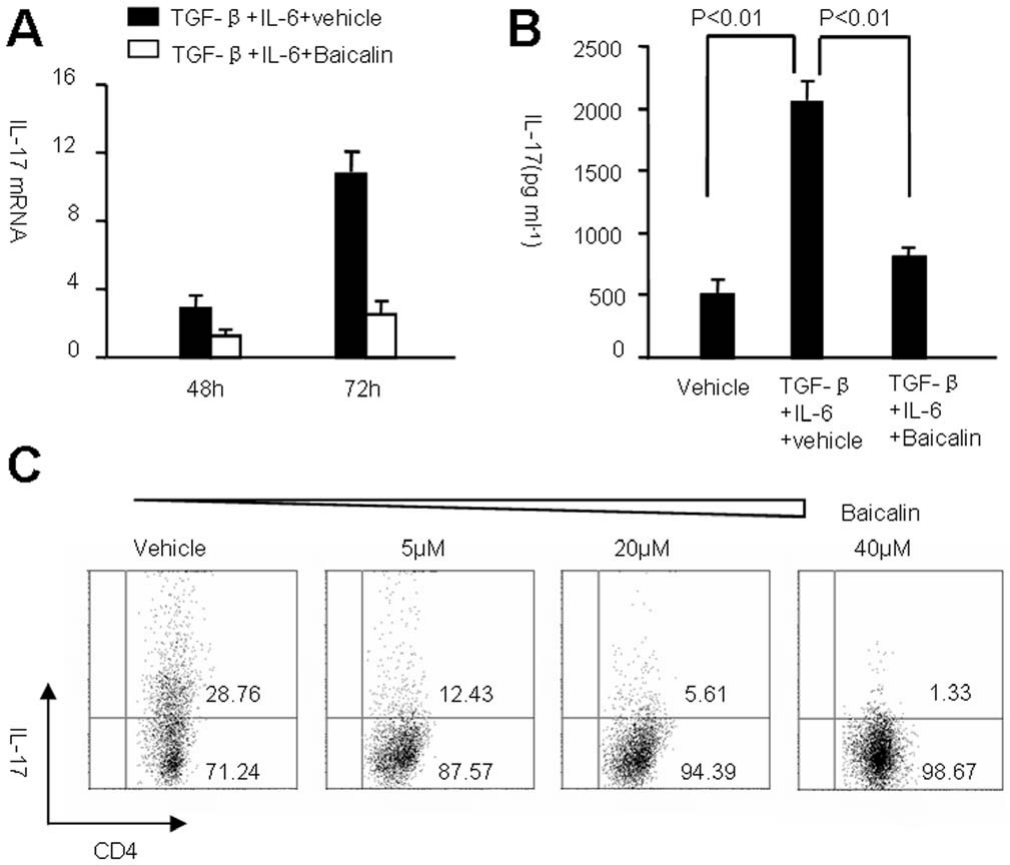
Baicalin inhibits T_H_17 cell differentiation in vitro. (**A**) CD4^+^CD25^−^ T cells from B6 mice were stimulated with anti-CD3, anti-CD28, and the indicated cytokines in the presence (open columns) or absence (filled columns) of Baicalin. IL-17 mRNA was analyzed by real-time RT-PCR at the indicated times. Results were expressed as mean ± SD, and fold induction compared with vehicle control (expression in vehicle control was set as 1.0). (**B**) IL-17 was examined by ELISA in culture supernatants after 3 days activation. Results were expressed as mean ± SD, and compared with vehicle control. (**C**) CD4^+^CD25^−^ T cells were cultured under T_H_17 conditions in the presence of indicated doses of Baicalin for 3 days, and then re-stimulated with PMA and Ionomycin in the presence of Brefeldin A for 5 hours. The percentages of IL-17^+^ cells among CD4^+^ T cells were examined by flow cytometry. These experiments were performed three times with similar results.

Baicalin (7-glucuronic acid, 5, 6-dihydroxyflavone, molecular weight  = 446.36. Figure S1A) is a flavonoid compound originally isolated from the Chinese Herb Huangqin (*Scutellaria* baicalensis Georgi). First, using 3-(4, 5-dimethylthiazol-2-yl)-2, 5-diphenyltetrazolium bromide (MTT) and flow cytometry, we observed that treatment with 20 µM Baicalin did not result in generalized inhibition of T cell proliferation and cell cycle (Figure S1B and C), thus 20 µM Baicalin was used in most in vitro experiments. To determine whether Baicalin controls the differentiation of T_H_17 cells, CD4^+^CD25^−^ T cells from B6 mice were isolated. Under T_H_17 culture conditions (TGF-β plus IL-6 stimulation), IL-17 mRNA expression was increased 2.9-fold compared to control cells on day 2 and 10.7-fold compared to control cells on day 3. Following addition of 20 µM Baicalin to the culture, IL-17 mRNA expression was inhibited. In fact, IL-17 expression was decreased to 1.2-fold on day 2 and 2.5-fold on day 3 compared to controls (Figure 1A). In addition, 20 µM Baicalin measurably inhibited IL-17 protein secretion (Figure 1B). We further proved that the suppression of T_H_17 cell differentiation was dependent on the dose of Baicalin (Figure 1C). These results provide evidence that Baicalin can suppress the development of T_H_17 cells.

### Baicalin inhibits IL-6 receptor and RORγt mRNA expression

**Figure 2:**
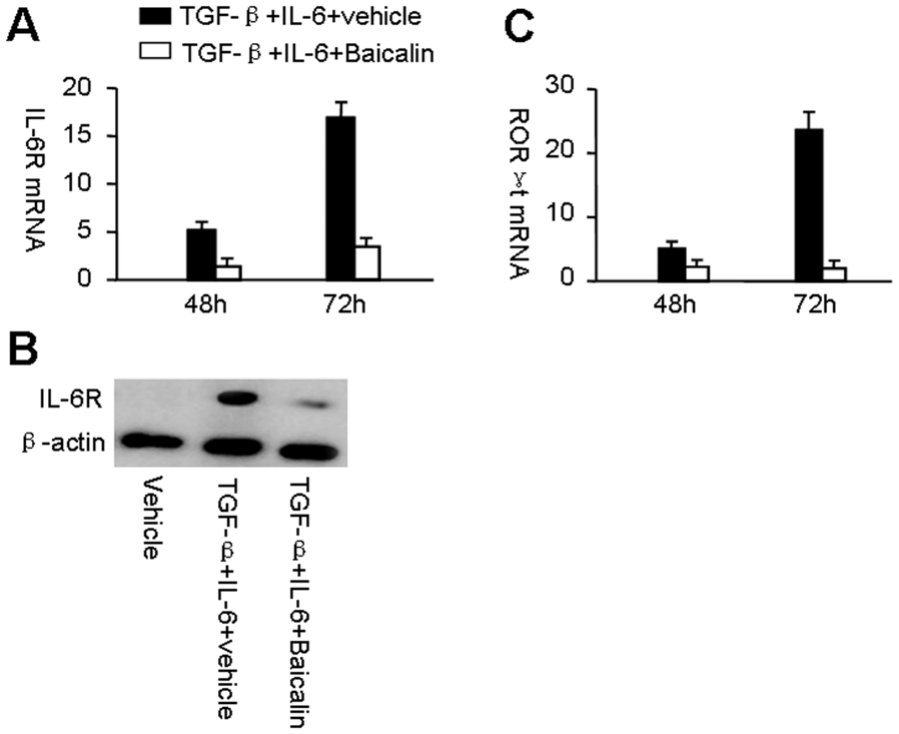
Baicalin inhibits IL-6R and RORγt mRNA expression. (**A**) CD4^+^CD25^−^ T cells from B6 mice were stimulated with anti-CD3, anti-CD28, and the indicated cytokines in the presence (open columns) or absence (filled columns) of Baicalin. IL-6R mRNA expression was analyzed by real-time RT-PCR at the indicated times. Results were expressed as mean ± SD, and fold induction compared with vehicle control (expression in vehicle control was set as 1.0). (**B**) CD4^+^CD25^−^ T cells were cultured under the indicated cytokines with or without 20 µM Baicalin for 2 days, IL-6R protein was measured by western blot analysis. (**C**) RORγt mRNA expression was analyzed by real-time RT-PCR at the indicated times. Results were expressed as mean ± SD, and fold induction compared with vehicle control (expression in vehicle control was set as 1.0). These experiments were performed three times with similar results.

IL-6, an acute-phase protein induced during inflammation, may “dictate” T_H_17 cell differentiation [26]. Thus, we next determine whether Baicalin-mediated inhibition of T_H_17 cell differentiation is IL-6-dependent. CD4^+^CD25^−^ T cells from B6 mice were stimulated with anti-CD3, anti-CD28, and the indicated cytokines in the presence or absence of Baicalin. IL-6 receptor (IL-6R) mRNA expression was analyzed by real-time RT-PCR at the indicated times. As expected, IL-6R mRNA was suppressed by Baicalin (Figure 2A). Further study confirmed that Baicalin could reduce IL-6R protein expression during Th17 cell differentiation (Figure 2B). RORγt, which is a key transcription factor involved in T_H_17 cell differentiation, is elicited by IL-6 and TGF-β [17]. During T_H_17 cell differentiation in vitro, addition of Baicalin reduced RORγt expression (Figure 2C). IL-23 expands the pool of T_H_17 cells [27], but Baicalin failed to affect the expression of IL-23 receptor during T_H_17 differentiation (Figure S2A). TGF-β and IL-21 can induce STAT3-mediated IL-17 expression during T_H_17 differentiation [16], while Baicalin did not restrain IL-21-induced STAT3 and IL-17 mRNA expression during T_H_17 cell differentiation (Figure S2B and 2C). These data suggest that Baicalin could reduce IL-6R and RORγt expression during T_H_17 cell differentiation, which imply that Baiclain might suppress de novo T_H_17 cell differentiation via inhibition of IL-6-mediated RORγt expression.

### Baicalin up-regulates Foxp3 and down-regulates RORγt-meidated IL-17 expression

**Figure 3:**
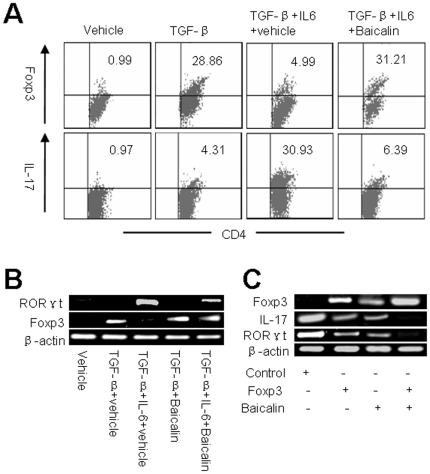
Baicalin up-regulates Foxp3 and down-regulates RORγt expression. (**A**) CD4^+^CD25^−^ T cells from B6 mice were stimulated with anti-CD3, anti-CD28, and the indicated cytokines, after 2 days stimulation, Baicalin was added for additional 2 days. The percentages of Foxp3^+^ and IL-17^+^ cells among CD4^+^ T cells were determined by flow cytometry. (**B**) CD4^+^CD25^−^ T cells from B6 mice were stimulated with anti-CD3, anti-CD28, and the indicated cytokines, after 2 days stimulation, Baicalin was added for additional 2 days. Foxp3 and RORγt mRNA expression were examined by RT-PCR. (**C**) CD4^+^CD25^−^ T cells from B6 mice were cultured under T_H_17 conditions for 2 days and then transiently transfected with control plasmids (Control) or Foxp3 expression plasmids (Foxp3) in the presence or absence of Baicalin. 2 days later, RORγt, IL-17, and Foxp3 mRNA were examined by RT-PCR.

TGF-β induces the differentiation of T_reg_ cells, whereas TGF-β in combination with IL-6 results in the differentiation of T_H_17 cells [19], [28]. CD4^+^CD25^−^ T cells from B6 mice were stimulated with anti-CD3, anti-CD28, and the indicated cytokines, after 2 days stimulation, 20 µM Baicalin was added for additional 2 days. Foxp3 and IL-17 intracellular expression in CD4^+^ T cells were determined by flow cytometry. Surprisingly, T cells cultured with Baicalin under conditions that otherwise promoted IL-6-dependent T_H_17 cell differentiation converted to Foxp3^+^ T cells with a concomitant decrease in T_H_17 cell differentiation (Figure 3A). In addition, Baicalin could inhibit RORγt-mediated IL-17 mRNA expression in established T_H_17 cells (Figure S3A). Thus, we hypothesized that Baicalin could inhibit RORγt transcriptional activity partly via up-regulation of endogenous Foxp3 expression, because previous report showed that Foxp3 could inhibit RORγt-mediated IL-17 expression and T_H_17 cell differentiation [29]. To support this hypothesis, we further showed that Baicalin in synergy with TGF-β could up-regulate endogenous Foxp3 expression in CD4^+^CD25^−^ T cells (Figure S3B), Baicalin could promote endogenous Foxp3 expression (Figure 3B, middle panel) and reduce RORγt expression during T_H_17 cell differentiation (Figure 3B, upper panel), and Baicalin together with forced expression of Foxp3 could inhibit RORγt and IL-17 mRNA expression during T_H_17 cell differentiation (Figure 3C). Collectively, these data imply that Baicalin could inhibit RORγt expression in established T_H_17 cells, together with up-regulating Foxp3 inhibit RORγt-mediated IL-17 expression.

### Baicalin inhibits T_H_17 cell differentiation in vivo

**Figure 4:**
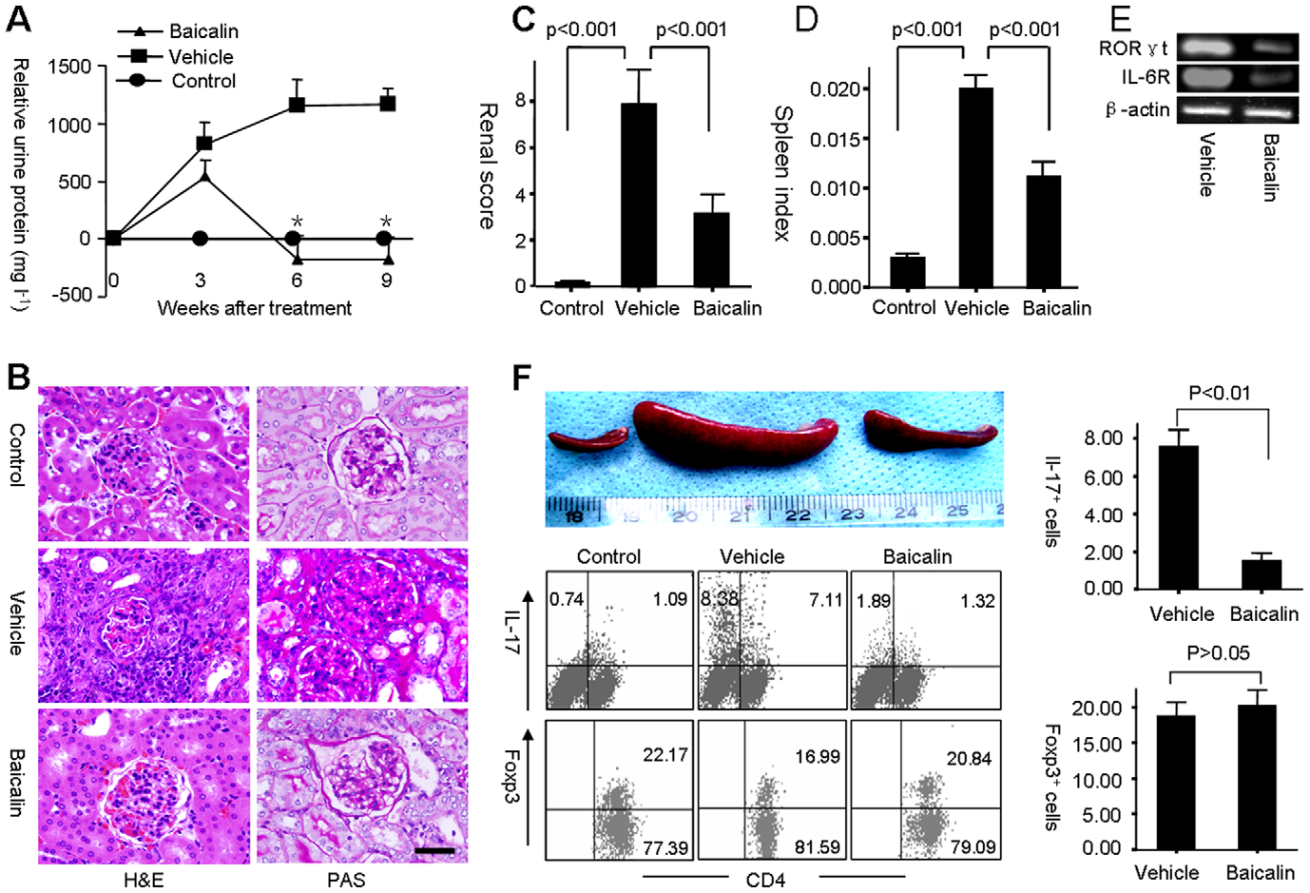
Baicalin inhibits T_H_17 cell differentiation in vivo. (**A**) MRL/lpr mice were treated with Baicalin or vehicle for 9 weeks, B6 mice (control) were treated with vehicle. Relative urine protein increases  =  urine protein (mg/L) at indicated time point - urine protein (mg/L) of week 0 (n = 6 for each group). In the comparison between Baicalin- and vehicle-treated mice, the asterisk indicates *p*<0.001. (**B**) Histopathology of kidneys. Hematoxylin and eosin staining (H&E, left) and periodic acid-Sciff-staining (PAS, right). Scale bar, 100 µm. (**C**) The renal score of control and MRL/lpr mice (n = 6 for each group). (**D**) Spleen index of control and MRL/lpr mice (n = 6 for each group). (**E**) IL-6R and RORγt mRNA levels in spleen cells of MRL/lpr mice were analyzed by RT-PCR. (**F**) The percentages of IL-17^+^ and Foxp3^+^ cells among CD4^+^ T cells of spleen cells isolated from MRL/lpr mice were determined by flow cytometry analysis. (n = 6 for each group). Results were expressed as mean ± SD. Spleens were shown.

To determine whether Baicalin controls the development of T_H_17 cells in vivo, lupus-prone MRL/lpr mice were treated with Baicalin or vehicle for nine weeks. Notably, mice without Baicalin treatment developed severe nephritis with increased urine protein, while mice receiving Baicalin were protected against nephritis with decreased urine protein (Figure 4A-C). Baicalin also protected the survival and liver function of MRL/lpr mice (Table 1 and Figure S4). Furthermore, Baicalin reduced the spleen index and inhibited differentiation of T_H_17 cells in spleens (Figure 4D-F). Interestingly, Baicalin only slightly affected the frequency of T_reg_ cells in vivo (Figure 4F).

**Table 1:** Survival data in MRL/lpr mice and B6 control.

Group	Survival
Control	6 of 6 (100%)
Vehicle	6 of 9 (66.7%)
Baicalin	6 of 7 (85.7%)

MRL/lpr mice were treated with Baicalin or vehicle for 9 weeks, B6 mice (control) were treated with vehicle.

**Figure 5:**
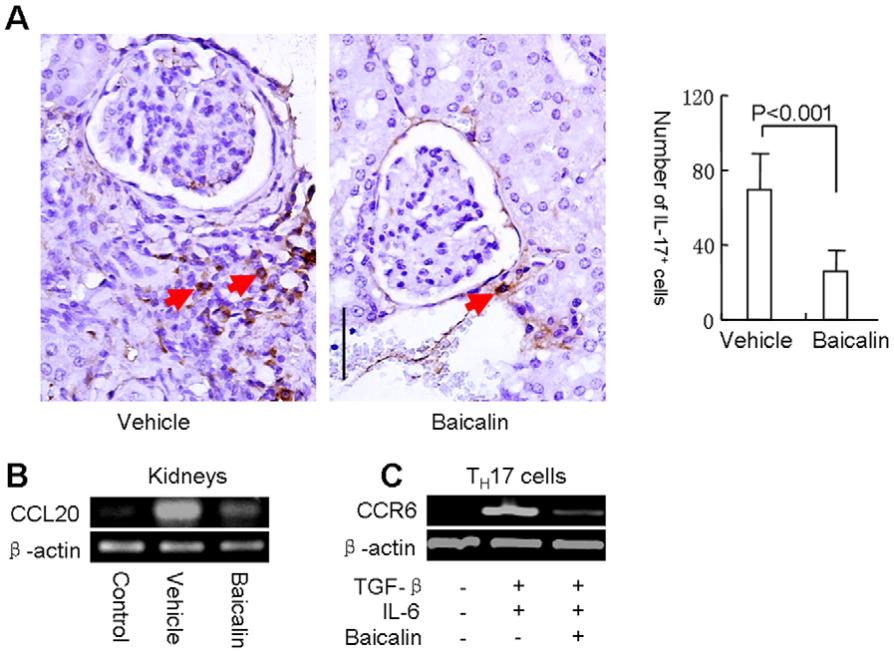
Baicalin inhibits IL-17^+^ lymphocyte infiltration into kidney. (**A**) IL-17 immunohistochemical staining in kidneys from MRL/lpr mice treated with Baicalin or vehicle for 9 weeks, arrow shows the typical IL-17^+^ lymphocytes. Scale bar, 100 µm. Five independent microscopic fields were selected randomly for each sample to ensure representativeness and homogeneity. Right panel shows the IL-17^+^ cells counted under ×100 magnification. (**B**) CCL20 mRNA expression in kidney. (**C**) CD4^+^CD25^−^ T cells from B6 mice were cultured under T_H_17 conditions with or without Baicalin for 3 days, CCR6 mRNA expression in T_H_17 cells was examined by RT-PCR. These experiments were performed three times with similar results.

Further study showed that Baicalin reduced the infiltration of T_H_17 cells into the kidneys (Figure 5A). Inflamed tissue produces CCL20 to facilitate the migration of CCR6-expressing T_H_17 cells to the inflamed tissues [30], [31]. Baicalin treatment inhibited CCL20 mRNA expression in kidneys and CCR6 expression in T_H_17 cells (Figure 5B and C), which indicated that Baicalin might interfere T_H_17 cell infiltration into kidneys via inhibition of the CCL20-CCR6 expression.

### Baicalin inhibits IL-17 mediated gene expression of inflammatory molecules

**Figure 6:**
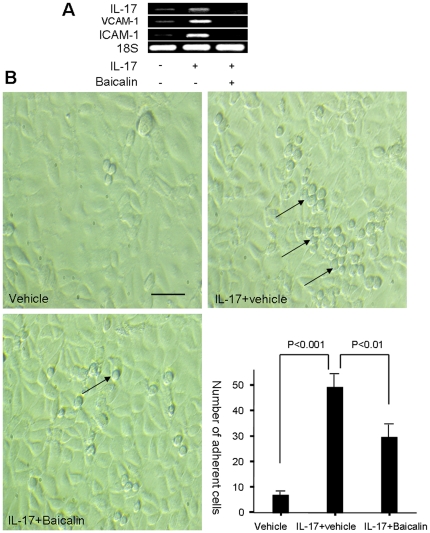
Baicalin inhibits IL-17 mediated gene expression of inflammatory molecules. (**A**) HUVEC was cultured in the presence of 50 ng/ml IL-17 in the presence or absence of Baicalin for 24 hours. Expression of the indicated genes was examined by RT-PCR. (**B**) HUVEC was stimulated in the presence of IL-17 with or without Baicalin for 24 hours. Jurkat cells were added for an additional 24 hours. After washing twice, the adhesive cells were counted under ×200 magnifications. Five independent microscopic fields were selected randomly for each sample to ensure representativeness and homogeneity. Results were expressed as mean ± SD. Arrows indicate the adhesive cells. Scale bar, 100 µm. These experiments were performed three times with similar results.

IL-17 acts as a potent inflammatory cytokine, and mediates leukocyte infiltration and tissue destruction [2], [32]. Baicalin inhibited expression of genes encoding inflammatory molecules (ICAM-1, VCAM-1, and IL-17) in HUVEC that were induced by exogenous IL-17 (Figure 6A). In support of these results, Baicalin reduced IL-17-induced adhesion of T cells to HUVEC (Figure 6B). In addition, Baicalin also suppressed gene expression of inflammatory mediators in MRL/lpr mouse kidney (Figure S5). Together, these data indicate that Baicalin could partially inhibit IL-17-induced inflammation.

## Discussion

Baicalin, which is a main active ingredient originally isolated from the root of Huangqin (*Scutellaria* baicalensis Georgi), has safety records in clinic and has been used as an anti-inflammatory drug in traditional Chinese medicine [21]. Baicalin has been found to possess anti-inflammatory, antioxidant and anti-allergic properties, and appears to contribute to the treatment of chronic inflammatory diseases, including hepatitis, allergic diseases, and EAE [22], [23], [33].

The binding of IL-6 with IL-6R plays a key role in the transcription of RORγt during the development of T_H_17 cells, and IL-6 blockade by treatment with an anti-IL-6R monoclonal antibody might inhibit the development of T_H_17 cells [34], [35]. IL-6-deficient mice do not express RORγt and IL-17 [17]. Together, these data suggest that IL-6 is a key cytokine to induce the expression of RORγt and the development of T_H_17 cells. Our data showed that Baicalin treatment inhibited the expression of IL-6R and RORγt under culture conditions promoting T_H_17 cell differentiation. However, the down-regulation of IL-6R was not accompanied by decreased expression of STAT3. IL-21 is also a key cytokine for STAT3-mediated T_H_17 cell differentiation [16], Baicalin did not suppress IL-21R and STAT3 mRNA expression induced by TGF-β and IL-6 (Figure S2A), which might explain that reduced expression of IL-6R was not accompanied by decreased mRNA expression of STAT3. Baicalin also hardly restrain IL-21-induced STAT3 and IL-17 mRNA expression during T_H_17 cell differentiation (Figure S2B and C). But Baicalin could affect the STAT3 phosphorylation induced by TGF-β and IL-6 (Figure S2D). Thus, these data implied that transcript levels of STAT3 did not mirror protein levels, and Baicalin might regulate T_H_17 cell differentiation by affecting STAT3 phosphorylation but not the expression of STAT3. Furthermore, cytokine receptors like IL-4R and IL-12Rβ2 have negative impacts on T_H_17 cell differentiation, our supplemental data showed that 20 µM Baicalin did not affect the mRNA expression of IL-4R and IL-12Rβ2 during T_H_17 cell differentiation (Figure S2A). These data implied that Baicalin might restrain de novo T_H_17 cell differentiation by abrogating IL-6 mediated RORγt transcription, and Baicalin-mediated inhibition of STAT3 activation might contribute to reduced STAT3-mediated gene expression, such as RORγt and IL-17A [36].

IL-23 receptor is important in the maturation of T_H_17 cells [14], [27], [37]. Although Baicalin did not affect IL-23 receptor mRNA expression during T_H_17 cell differentiation, Baicalin, when added 2 days after initiation of T_H_17 cultures, also inhibited differentiation of T_H_17 cells. We first confirmed that Baicalin could inhibit RORγt-mediated IL-17 mRNA expression in established T_H_17 cells (Figure S3A), which might partly stem from the block in IL-6R signaling in T_H_17 cells. We further detected that Baicalin not only inhibited IL-17 expression but also up-regulated Foxp3 expression (Figure 3A), thus we hypothesized that Baicalin-induced Foxp3 expression might exert inhibition on RORγt-mediated IL-17 expression during T_H_17 cell differentiation. Because previous study has proved that Foxp3 could interact with RORγt and inhibit RORγ-directed IL-17 expression during T_H_17 cell differentiation [29]. To support this hypothesis, we showed that Baicalin together TGF-β could up-regulate endogenous Foxp3 mRNA and down-regulate RORγt mRNA expression (Figure 3A, B, and Figure S3B). In addition, exogenous over expressed Foxp3 could inhibit RORγt-mediated IL-17 mRNA expression in T_H_17 cells, Baicalin together with Foxp3 might augment inhibition of IL-17 mRNA expression (Figure 3C). Interestingly, we also noticed that RORγt mRNA expression was also inhibited by forced expression of Foxp3, and Baicalin together with exogenous Foxp3 have a additive inhibition of RORγt expression (Figure 3C). Whereas further study should be performed to make clear the mechanism of Foxp3-mediated inhibition of RORγt expression. All together, these data implied that Baicalin could up-regulate Foxp3 expression and suppress RORγt-mediated IL-17 expression in established T_H_17 cells.

In our study, we unexpectedly found that Baicalin not only inhibited the differentiation of T_H_17 cells but also promoted TGF-β-mediated differentiation of T_reg_ cells in vitro. Thus, Baicalin appeared to play a dual role in T-cell differentiation by mediating a reciprocal balance of Foxp3 and RORγt. IL-6 is a key cytokine to inhibit Foxp3 expression during T_reg_ cell differentiation [19], inhibition of IL-6R expression could increase T_reg_ cell differentiation [38] Thus, Baicalin might induce Foxp3 expression by restoring IL-6-mediated inhibition of Foxp3 expression in vitro. In contrast to the observation that Baicalin enhanced Foxp3 expression in vitro, Baicalin treatment in MRL/lpr mice only slightly affected CD4^+^Foxp3^+^ T cells. This minor expansion of Foxp3^+^ T cells might stem from the strong inhibition of excessive inflammatory cytokines and lack of TGF-β in vivo [38], [39], [40]. Although we observed that 20 µM Baicalin did not affect cytokines expression during the differentiation of T_H_1 and T_H_2 cells in vitro (Figure S6), further study should be done to explore different concentrations of Baicalin on the differentiation of T_H_1 and T_H_2 cells.

The number of T_H_17 cells was found to be increased in murine model of SLE, including BXD_2_ [41], SNF_1_ [42], NZB×NZW F_1_ [43], [44], and Ro52 knockout mice [45]. Our previous studies, as well as others, showed that there were expansion of T_H_17 cells in MRL/lpr mice [9], [46], [47]. Our unpublished data also showed that treatment with anti-IL-17 antibody could protect MRL/lpr mice against disease onset. Together, these data suggested that T_H_17 cells might play a key role in the pathogenesis of MRL/lpr mice. Here we observed that Baicalin could reduce IL-6R and RORγt mRNA expression in spleens of MRL/lpr mice (Figure 4E), and Baicalin could accordingly inhibit T_H_17 cell differentiation in vivo (Figure 4F). These results were consistent with in vitro study of Baicalin on the differentiation of T_H_17 cells. Interestingly, we noticed that a high percentage of IL-17 producers was CD4^−^ cells in MRL/lpr mice (Figure 4F). Actually, T_H_17 cells (CD4^+^IL-17^+^ T cells) are main source of IL-17 during chronic inflammatory responses. However, in mice other subsets can also express IL-17, including CD8^+^ T cells, invariant natural killer T (NKT) cells, and γδ T cells [48], [49], [50], [51]. Thus, we hypothesized that CD4^−^L-17^+^ cells were also expanded in MRL/lpr mice duo to severe inflammatory responses, but further study should be performed to dissect the specific source and function of these groups of CD4^−^L-17^+^ cells.

Our data showed that Baicalin not only inhibited the differentiation of T_H_17 cells in spleen but also reduced the infiltration of T_H_17 cells into kidney, which might result from inhibition of the CCL20-CCR6 signaling pathway, since expression of CCL20 and CCR6 mRNA was found to be down-regulated by Baicalin. IL-17 is a key T_H_17 cell-derived cytokine, which is implicated in leukocyte recruitment [32]. Treatment with Baicalin could suppress production of IL-17-mediated adhesion of T cells to HUVEC. Our in vivo studies further confirmed that Baicalin could inhibit IL-17-related inflammatory mediators, such as IL-22, IL-1, TNF-α, VCAM-1, and ICAM-1 (Figure S5A). Through the potent inhibition of these adhesion molecules and inflammatory mediators, Baicalin might further impede the recruitment of T_H_1, T_H_2 or other effector cells in vivo. Thus, we are not making conclusions on the observed inhibition of T_H_17 cells as the only function of Baicalin in vivo. Further study should be done to dissect the other specific subsets of effector cells affected by Baicalin in MRL/lpr mice. In addition, our unpublished data also showed that Baicalin could inhibit T_H_17 cell differentiation in complete Freund's adjuvant induced inflammatory arthropathy mice, and ovalbumin immunized mice. Together, these data suggest that Baicalin could inhibit T_H_17 cell differentiation in vivo, and exert therapeutic effects via inhibition of IL-17-mediated inflammation.

Our findings define a role of Baicalin in T_H_17 lineage commitment, thereby linking this natural compound to adaptive immunity in a way that has important implications for immune homeostasis and inflammatory diseases. Taken together, these findings suggest that Baicalin might be a promising therapeutic agent for the treatment of T_H_17 cells-mediated inflammatory diseases.

## Methods

### Plasmids, cell lines, and transfection

The Foxp3-IRES-GFP expression plasmid (pZIGF) and control plasmid were kindly provided by Wang Shengjun. T_H_17 cells were transiently transfected by electroporation according to the manufacturer's protocol (Eppendorf, Hamburg, Germany). 48 hours after transfection, RORγt, IL-17, and Foxp3 mRNA expression were examined by RT-PCR. Human umbilical vein endothelial cell (HUVEC) line was maintained in Dulbecco's modified Eagle's medium (DMEM; Hyclone, Logan, UT). Jurkat cells were maintained in RPMI 1640 medium (Gibco, Grand Island, NY).

### Mice and histopathology

C57BL/6 (B6) and lupus-prone MRL/lpr mice were purchased from the Shanghai Laboratory Animal Center (Chinese Academy of Sciences). The animal study was approved by the institutional animal care and use committee of Zhongshan Hospital, Fudan University (ZS0862701). All mice were maintained under pathogen-free conditions. The onset of autoimmune diseases in MRL/lpr mice was monitored by the assessment of proteinuria. After clinical onset of disease, Baicalin (100 mg/kg; Purity>98%, National Institute for the Control of Pharmaceutical and Biological Products, Beijing, China; Baicalin was dissolved in phosphate-buffered saline prior to experimentation.) or PBS vehicle was given intraperitoneally every day for 9 weeks. For detection of urine protein, the total urine of 24 h were first collected, and performed according to the manufacturer's directions (Roche). Relative urine protein increases  =  urine protein (mg/L) at indicated time point - urine protein (mg/L) of week 0. At the time of sacrifice (9 weeks after treatment), the kidneys were fixed with formaldehyde, embedded in paraffin, stained with hematoxylin and eosin (H&E), and IL-17 (Santa Cruz Biotechnology, CA).

The slides were read and interpreted in a blinded fashion, grading the kidneys for glomerular inflammation, proliferation, crescent formation, and necrosis. Interstitial changes and vasculitis were also noted. Scores from 0 to 3 were assigned for each of these features and then added together to yield a final renal scores. For example, glomerular inflammation was graded: 0, normal; 1, few inflammatory cells; 2, moderate inflammation; and 3, severe inflammation. Detailed pathological assessment was performed as described previously [52]. The spleens of MRL/lpr mice were collected to calculate the spleen index. Spleen index  =  spleen weight (g) divided by body weight (g).

### CD4^+^ T cell isolation, culture conditions, and western blot

CD4^+^CD25^−^ T cells from B6 mice spleens were isolated by fluorescence activated cell sorting (FACS). T cells were stimulated with 2 µg/ml plate-bound anti-CD3e (clone:17A2) and 2 µg/ml soluble anti-CD28 (clone: 37.51; eBioscience, San Diego, CA) for the indicted number of days. Where indicated, cultures were supplemented with 5 ng/ml TGF-β, 20 ng/ml IL-6 (PeproTech, RockyHill, NJ), and 20 µM Baicalin (Baicalin was dissolved in DMSO), and DMSO vehicle control was used in some experiments. For some experiments, CD4^+^CD25^−^ T cells were cultured under T_H_17 culture conditions (TGF-β plus IL-6 stimulation) for 2 days, and then transfected with Foxp3 expression plasmids by electroporation. For detection of IL-6R protein, CD4^+^CD25^−^ T cells were cultured under T_H_17 culture conditions with or without 20 µM Baicalin for 2 days, monoclonal anti-IL-6R was used to detect the protein expression of IL-6R (Santa Cruz), and β-actin was used as internal control (Santa Cruz).

### Intracellular cytokine staining and flow cytometry analysis

For detection of T_H_17 cells, cells obtained from in vitro cultures or spleen cells from mice were incubated for 5 hours with 50 ng/ml phorbol myristate acetate (PMA) and 750 ng/ml ionomycin in the presence of 20 µg/ml brefeldin A (Sigma-Aldrich) in a tissue culture incubator at 37°C. Surface staining with FITC-conjugated anti-CD4 (eBioscience) was first performed for 15 min, then cells were re-suspended in Fixation/Permeabilization solution according to the manufacturer's instructions (Invitrogen), intracellular staining of PE-conjugated anti-IL-17 or isotype control was performed according to the manufacturer's protocol (eBioscience). After staining, we first gated on CD4^+^ T cells, then CD4^+^IL-17^+^ cells were analyzed in a CD4^+^ gate in a FACS-Calibur (BD-Bioscience, Biosciences, San Jose, CA), and followed by analysis with FlowJo software (Tree Star, San Carlos, CA).

For detection of T_reg_ cells, cells were treated according to the Foxp3-staining kit protocol (eBioscience). Gating was on CD4^+^ T cells first, and then Foxp3^+^ cells were analyzed in CD4^+^ gate.

### Cytokine production

Sorted CD4^+^CD25^−^ T cells from B6 mice were cultured under neutral conditions or in the presence of 5 ng/ml TGF-β plus 20 ng/ml IL-6 with or without 20 µM Baicalin for 3 days. IL-17 concentrations were determined by ELISA (R&D Systems, Minneapolis, MN).

### HUVEC and T cell co-culture

HUVEC was seeded into 12-well plates (10,000 cells/well) and allowed to adhere for 24 hours. Then HUVEC was stimulated with 50 ng/ml IL-17 (eBioscience) for 24 hours with or without 20 µM Baicalin. After stimulation, Jurkat cells were added to the HUVEC cultures at a 1∶5 ratio, and the co-culture was extended for an additional 24 hours. The HUVEC was then washed twice to eliminate the non-adherent Jurkat cells. The adhesion of T cells was counted under ×200 magnification.

### RNA isolation and real-time RT-PCR

Total RNA was prepared with the use of the Trizol reagent (Invitrogen). The cDNA was synthesized with a first-strand cDNA synthesis kit and oligo (dT) primers (Fermentas, Hanover, MD), and gene expression was examined with a Bio-Rad iCycler Optical System (Bio-Rad, Richmond, CA) using a SYBR green real-time PCR Master Mix (Toyobo, Osaka, Japan). The 2^−ΔΔCt^ method was used to normalize transcription to human 18S or mus β-actin and calculate fold induction relative to controls. The primer pairs could be seen in Table 2.

**Table 2:** The following primer pairs were used.

Gene	Forward (5′-3′)	Reverse (5′-3′)
Mus RORγt	CCGCTGAGAGGGCTTCAC	TGCAGGAGTAGGCCACATTACA
Mus β-actin	GACGGCCAGGTCATCACTATTG	AGGAAGGCTGGAAAAGAGCC
Mus Foxp3	CCCAGGAAAGACAGCAACCTT	TTCCACAACAAGGCCACTTG
Mus IL-17A	CTCCAGAAGGCCCTCAGACTCA	GGGTCTTCATTGCGGTGG
Mus IL-6 receptor	GCAAGTCCAGCCACAACG	ACTCGGGTCCCAGGTCTCA
Mus CCR6	GTGGTGTATGAGAAGGAAGAATAAGATG	GTCTGCCTGGAGATGTAGCTTTC
Mus CCL20	CTGATGCTTTTTTGGGATGGA	CCCCAGCTGTGATCATTTCC
Human ICAM-1	ATCTGTGTCCCCCTCAAAAG	GGTCTCTATGCCCAACAACT
Human VCAM-1	TACAACCGTCTTGGTCAGCC	CCACAGGATTTTCGGAGCA
Human 18S	GCCCGAAGCGTTTACTTTGA	TCCATTATTCCTAGCTGCGGTATC
Human IL-17	AAAGTGGCCCGGATGTGAGA	GACATTGTGCCCTGCCCTTCT

### Statistical analysis

The quantitative data were expressed as the means ± standard deviation (SD). The statistical significance was determined by ANOVA followed by Bonferroni post-hoc test for multiple comparisons or the Student's *t*-test. A paired t-test was also used in some cases. All *p* values ≤0.05 were considered significant.

## Supporting Information

Figure S1 **Baicalin does not inhibit T cell proliferation.** (**A**) Chemical structure of Baicalin. (**B**) FACS-sorted naïve CD4^+^CD25^−^ T cells from B6 mice were stimulated with anti-CD3 and anti-CD28 in the presence of indicated doses of Baicalin for 3 days. Cell proliferation was measured by MTT. (**C**) Cell cycle was analyzed by flow cytometry. These experiments were performed three times with similar results.
(TIF)

Figure S2 **Baiclain does not affect IL-23R, IL-21R, and other cytokines mRNA expression.** (**A**) FACS-sorted CD4^+^CD25^−^ T cells from B6 mice were stimulated with TGF-β and IL-6 in the presence of absence of Baicalin for 2 days. IL-23R, IL-21R, STAT3, IL-4R, and IL-12Rβ2 mRNA expression were examined by RT-PCR. (**B**) FACS-sorted CD4^+^CD25^−^ T cells from B6 mice were stimulated with TGF-β and IL-21 in the presence or absence of Baicalin for 2 days. STAT3, IL-17, and Foxp3 mRNA expression were examined by RT-PCR. (**C**) FACS-sorted CD4^+^CD25^−^ T cells from B6 mice were stimulated with TGF-β and IL-21 in the presence or absence of Baicalin for 3 days, IL-17 and STAT3 mRNA expression were examined by real-time RT-PCR. (**D**) FACS-sorted CD4^+^CD25^−^ T cells from B6 mice were stimulated with TGF-β and IL-6 in the presence or absence of Baicalin for 24h, Stat3 phosphorylation was analyzed by FACS. These experiments were performed three times with similar results.
(TIF)

Figure S3 **Baicalin promotes Foxp3 mRNA and inhibit RORγt-mediated IL-17 mRNA expression in vitro.** (**A**) CD4^+^CD25^−^ T cells from B6 mice were cultured under T_H_17 conditions for 2 days and then transiently transfected with control plasmids (Control) or RORγt expression plasmids (RORγt) in the presence or absence of Baicalin. 2 days later, IL-17 mRNA was examined by RT-PCR. (**B**) FACS-sorted CD4^+^CD25^−^ T cells from B6 mice were stimulated with TGF-β and/or Baicalin for 3 days. Foxp3 mRNA expression was examined by real-time RT-PCR. Results were expressed as mean ± SD, and fold induction compared with vehicle control (expression in vehicle control was set as 1.0). These experiments were performed three times with similar results.
(TIF)

Figure S4 **Baicalin protects the liver function of MRL/lpr mice.** (**A**) MRL/lpr mice were treated with Baicalin or vehicle for 9 weeks, B6 mice (control) were treated with vehicle. Alanine aminotransferase (ALT) in serum was checked (n = 6 for each group). Results were expressed as mean ± SD. (**B**) Aspartate aminotransferase (AST) in serum was checked (n = 6 for each group). Results were expressed as mean ± SD.
(TIF)

Figure S5 **Baicalin inhibits the gene expression of inflammatory mediators in vivo.** The gene expression of inflammatory mediators in kidneys of MRL/lpr mice treated with Baicalin or vehicle for 9 weeks was analyzed by RT-PCR. These experiments were performed three times with similar results.
(TIF)

Figure S6 **20µM Baicalin does not affect IFN-γ and IL-4 mRNA expression.** CD4^+^CD25^−^ T cells from B6 mice were stimulated with anti-CD3, anti-CD28, and the indicated cytokines in the presence or absence of Baicalin for 3 days. IFN-γ and IL-4 mRNA expression were analyzed by RT-PCR. These experiments were performed three times with similar results.
(TIF)
