# Supplementary figures and images for: Identification of Baicalin as an Immunoregulatory Compound by Controlling TH17 Cell Differentiation
Source: PLoS One. 2011 Feb 16;6(2):e17164. doi: 10.1371/journal.pone.0017164 (PMC3040219; doi:10.1371/journal.pone.0017164)

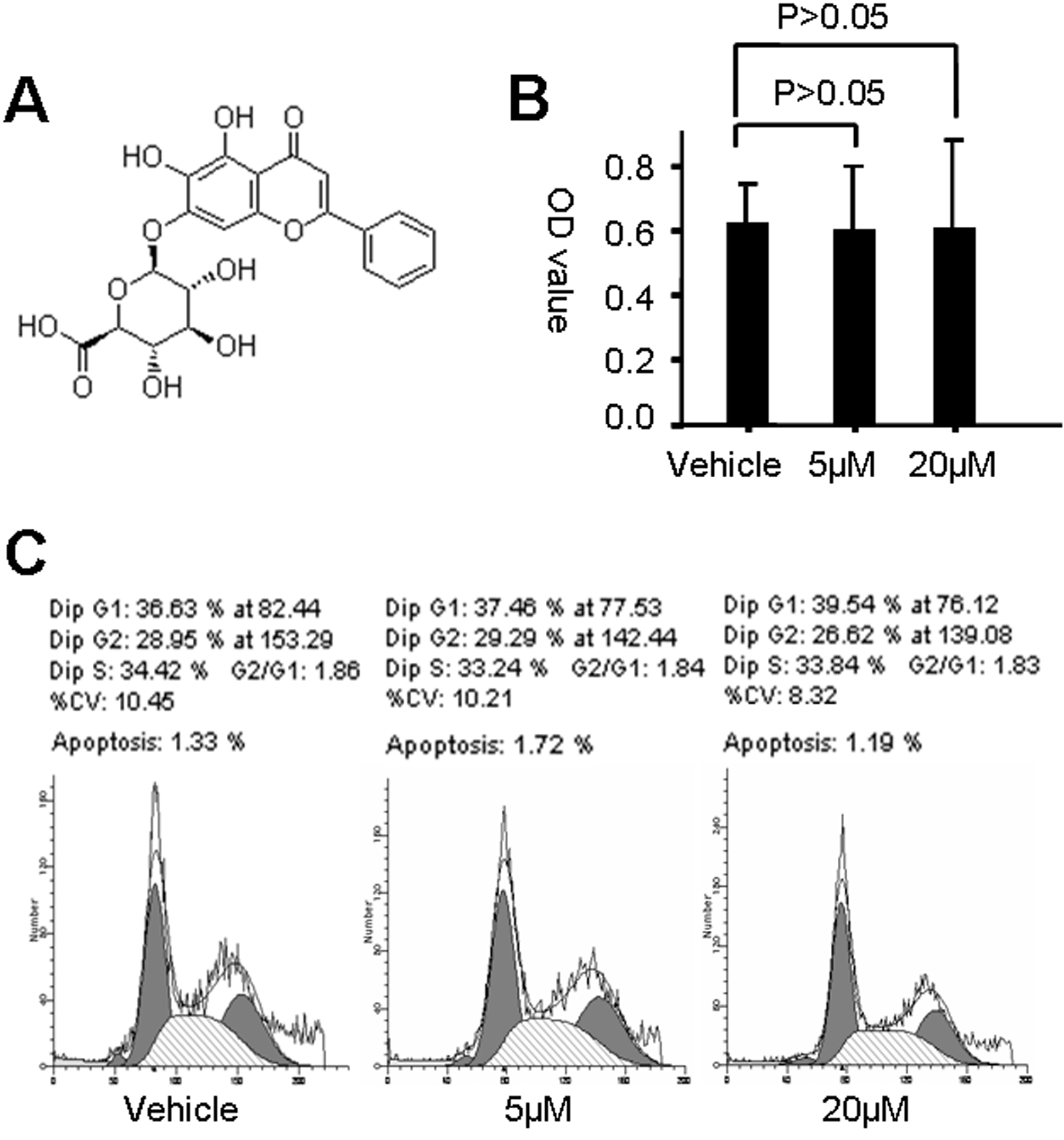

Supplement: Figure S1 — Baicalin does not inhibit T cell proliferation. (A) Chemical structure of Baicalin. (B) FACS-sorted naïve CD4+CD25− T cells from B6 mice were stimulated with anti-CD3 and anti-CD28 in the presence of indicated doses of Baicalin for 3 days. Cell proliferation was measured by MTT. (C) Cell cycle was analyzed by flow cytometry. These experiments were performed three times with similar results. (TIF) [file pone.0017164.s001.tif]

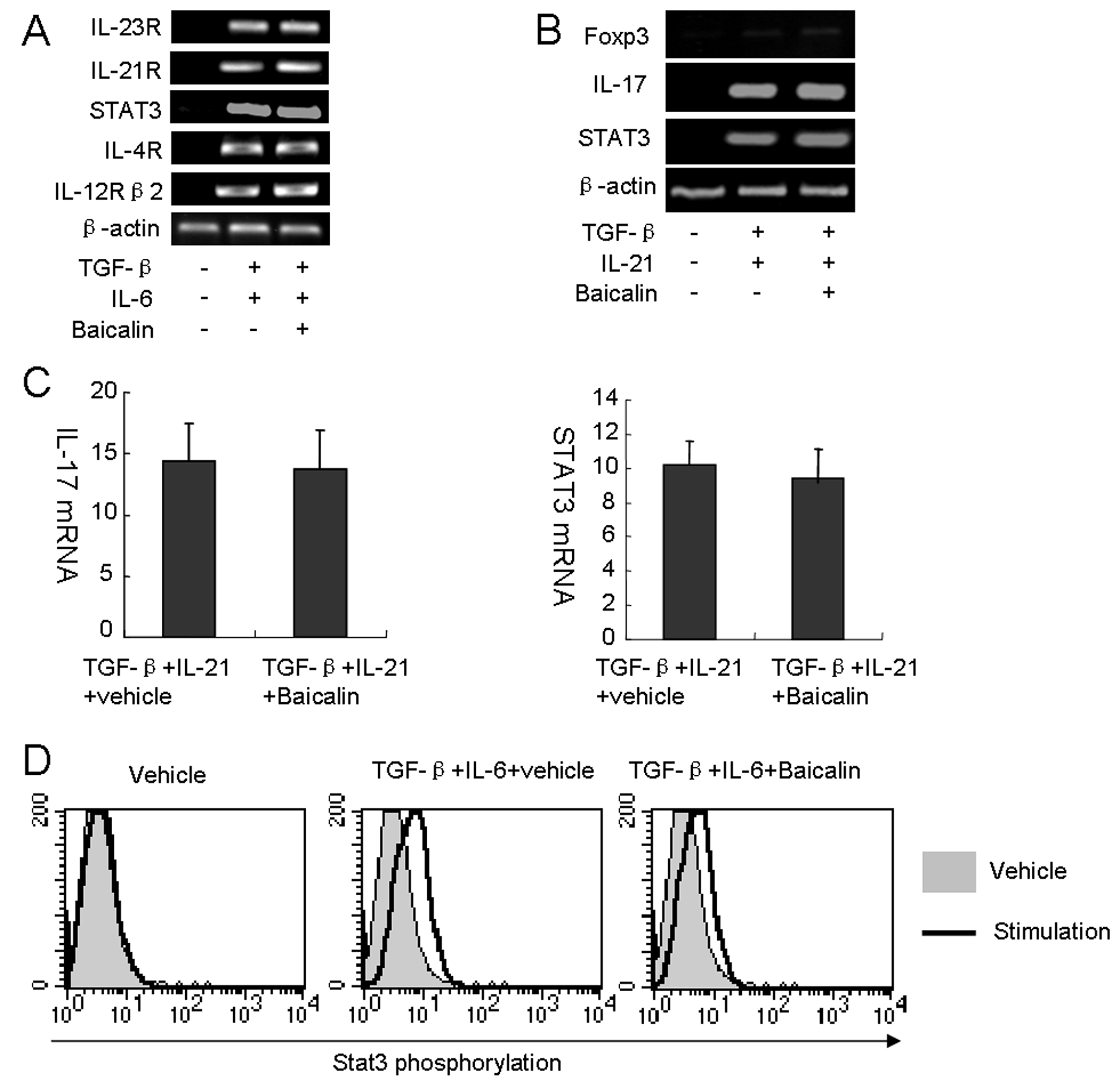

Supplement: Figure S2 — Baiclain does not affect IL-23R, IL-21R, and other cytokines mRNA expression. (A) FACS-sorted CD4+CD25− T cells from B6 mice were stimulated with TGF-β and IL-6 in the presence of absence of Baicalin for 2 days. IL-23R, IL-21R, STAT3, IL-4R, and IL-12Rβ2 mRNA expression were examined by RT-PCR. (B) FACS-sorted CD4+CD25− T cells from B6 mice were stimulated with TGF-β and IL-21 in the presence or absence of Baicalin for 2 days. STAT3, IL-17, and Foxp3 mRNA expression were examined by RT-PCR. (C) FACS-sorted CD4+CD25− T cells from B6 mice were stimulated with TGF-β and IL-21 in the presence or absence of Baicalin for 3 days, IL-17 and STAT3 mRNA expression were examined by real-time RT-PCR. (D) FACS-sorted CD4+CD25− T cells from B6 mice were stimulated with TGF-β and IL-6 in the presence or absence of Baicalin for 24h, Stat3 phosphorylation was analyzed by FACS. These experiments were performed three times with similar results. (TIF) [file pone.0017164.s002.tif]

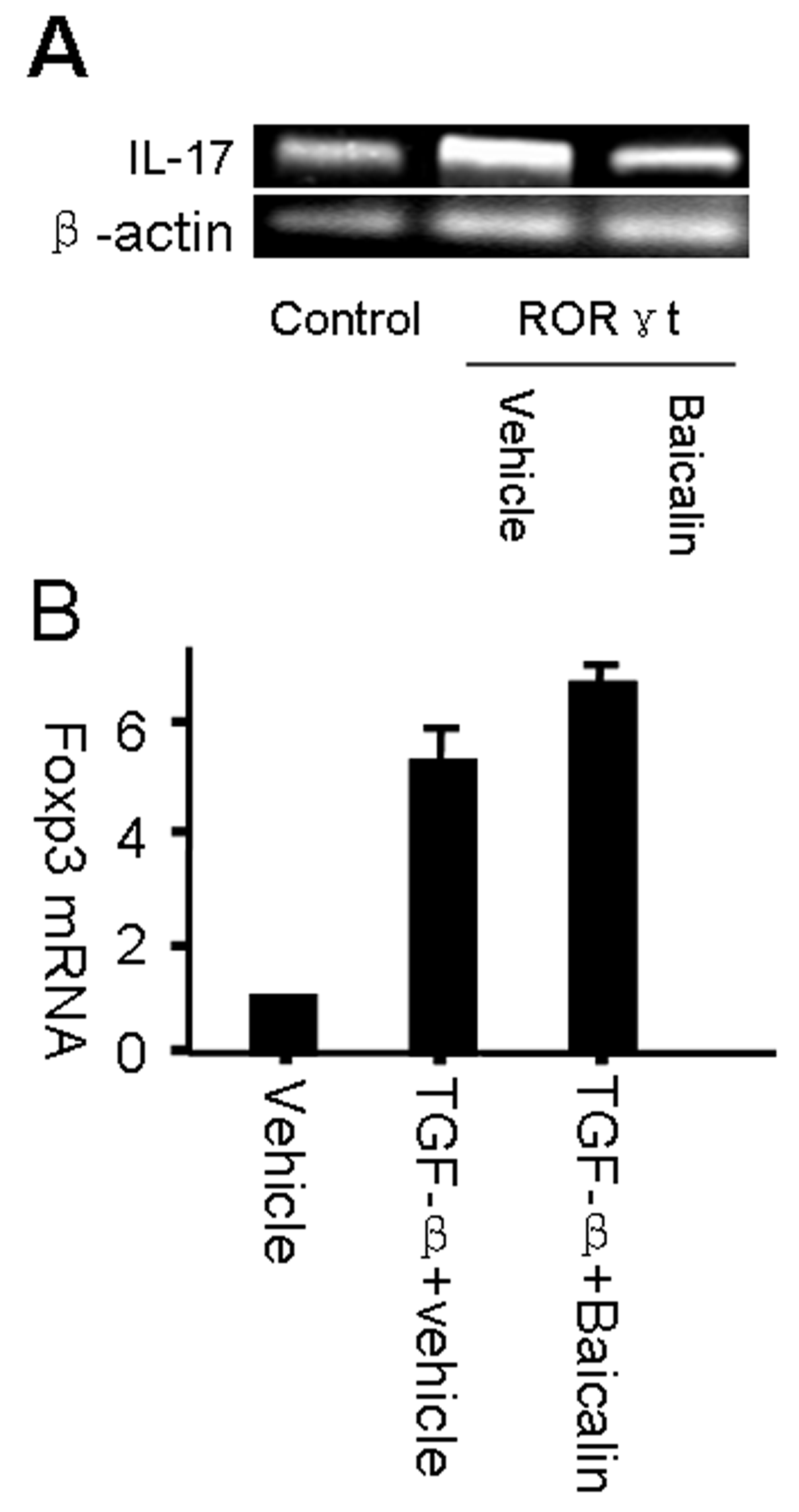

Supplement: Figure S3 — Baicalin promotes Foxp3 mRNA and inhibit RORγt-mediated IL-17 mRNA expression in vitro. (A) CD4+CD25− T cells from B6 mice were cultured under TH17 conditions for 2 days and then transiently transfected with control plasmids (Control) or RORγt expression plasmids (RORγt) in the presence or absence of Baicalin. 2 days later, IL-17 mRNA was examined by RT-PCR. (B) FACS-sorted CD4+CD25− T cells from B6 mice were stimulated with TGF-β and/or Baicalin for 3 days. Foxp3 mRNA expression was examined by real-time RT-PCR. Results were expressed as mean ± SD, and fold induction compared with vehicle control (expression in vehicle control was set as 1.0). These experiments were performed three times with similar results. (TIF) [file pone.0017164.s003.tif]

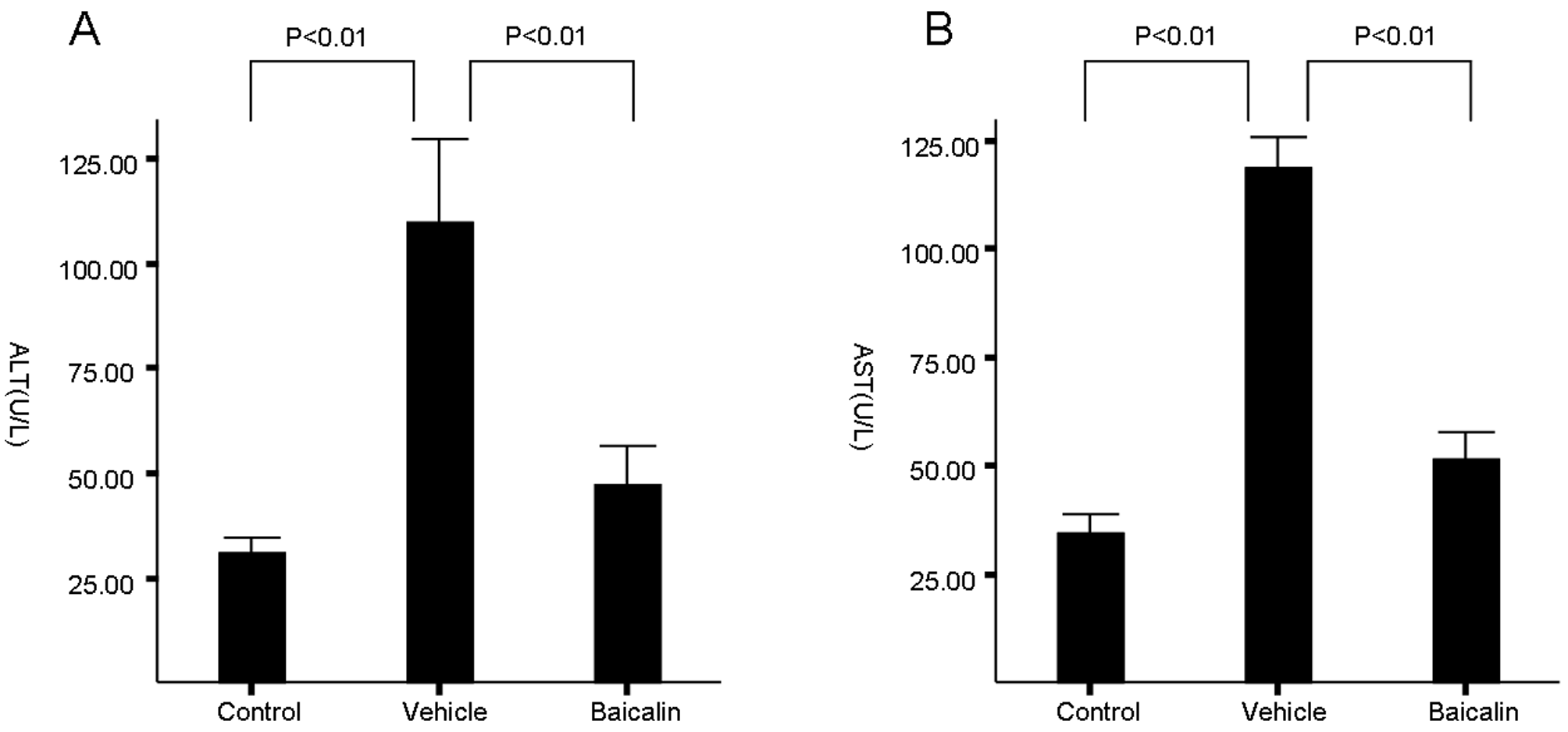

Supplement: Figure S4 — Baicalin protects the liver function of MRL/lpr mice. (A) MRL/lpr mice were treated with Baicalin or vehicle for 9 weeks, B6 mice (control) were treated with vehicle. Alanine aminotransferase (ALT) in serum was checked (n = 6 for each group). Results were expressed as mean ± SD. (B) Aspartate aminotransferase (AST) in serum was checked (n = 6 for each group). Results were expressed as mean ± SD. (TIF) [file pone.0017164.s004.tif]

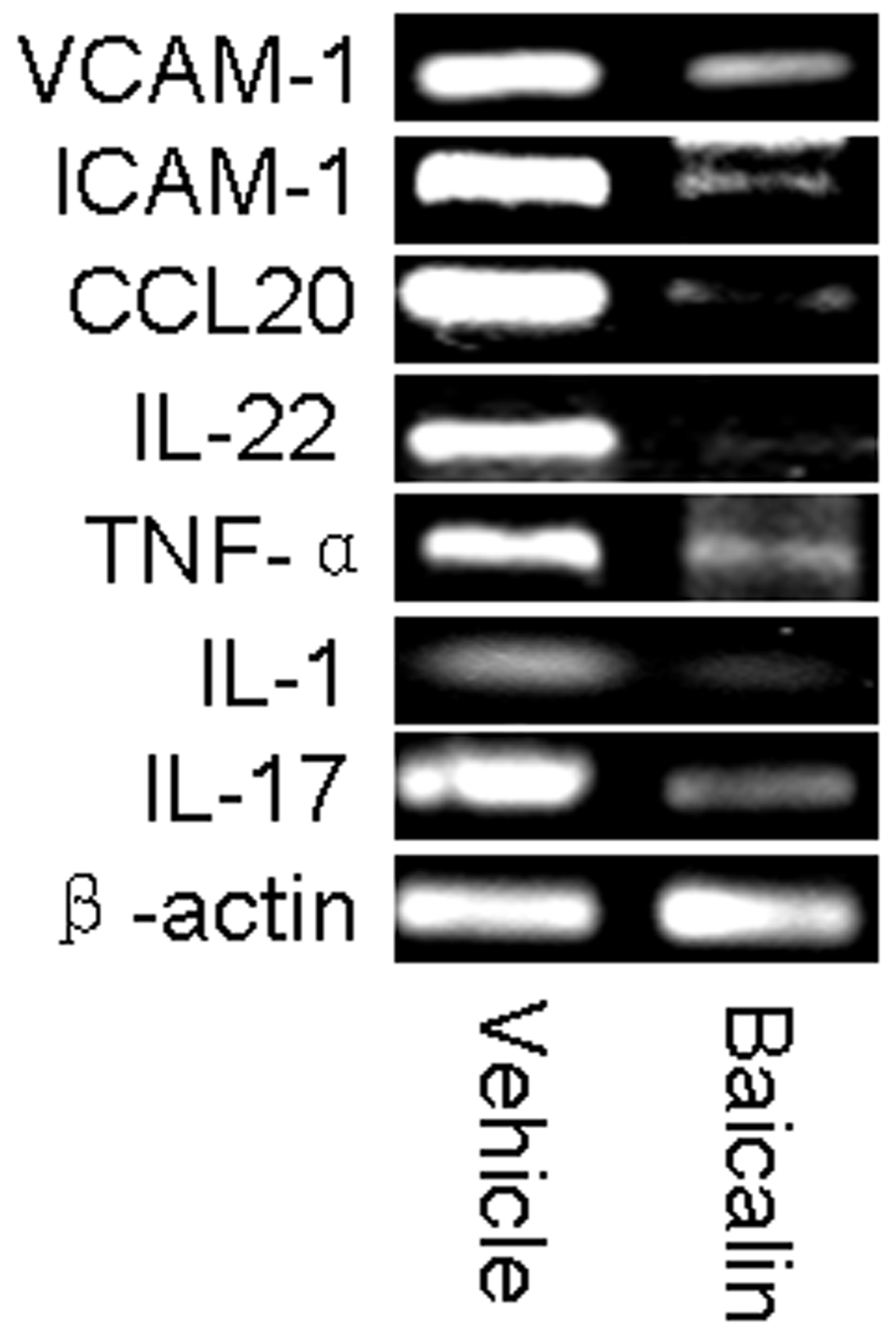

Supplement: Figure S5 — Baicalin inhibits the gene expression of inflammatory mediators in vivo. The gene expression of inflammatory mediators in kidneys of MRL/lpr mice treated with Baicalin or vehicle for 9 weeks was analyzed by RT-PCR. These experiments were performed three times with similar results. (TIF) [file pone.0017164.s005.tif]

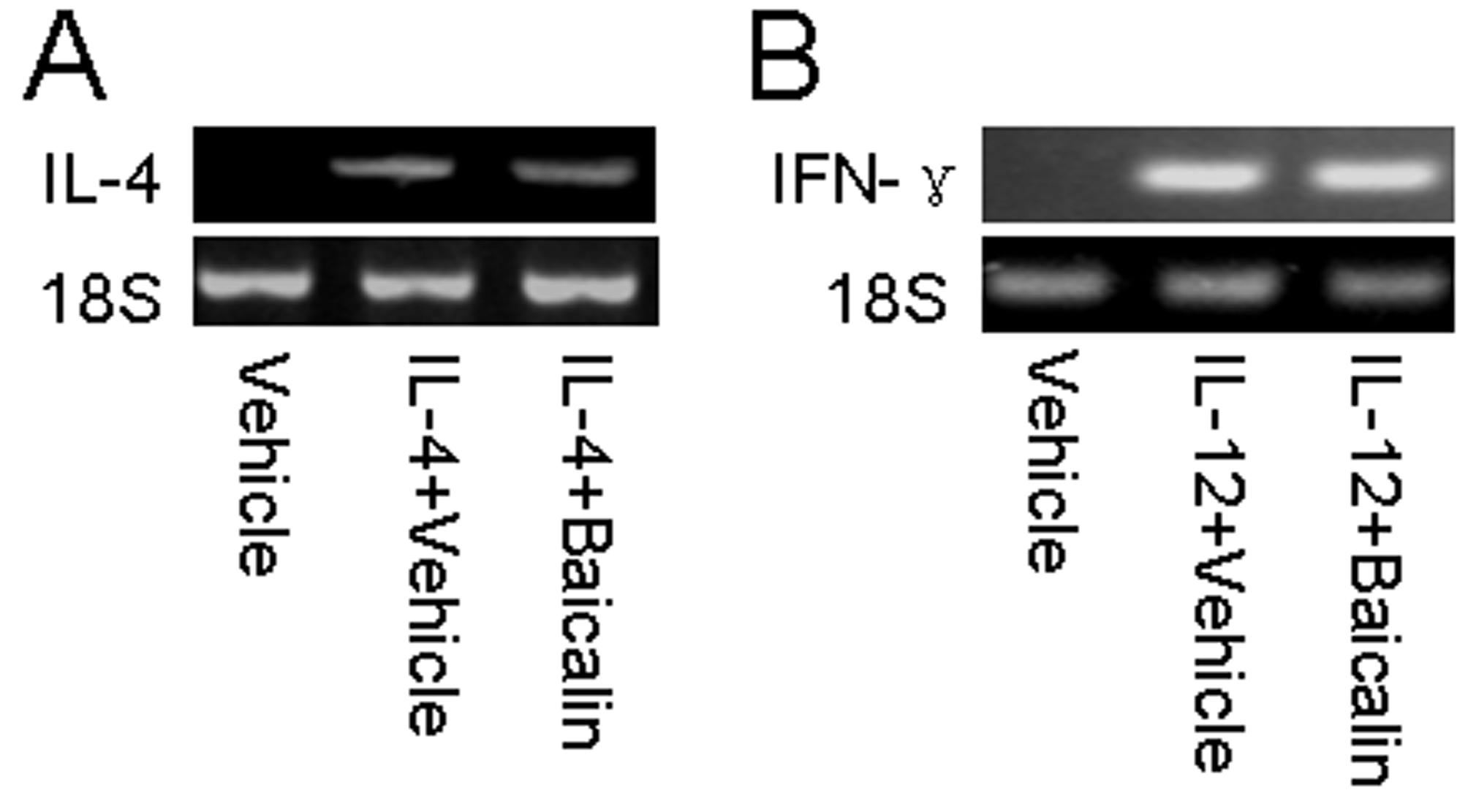

Supplement: Figure S6 — 20µM Baicalin does not affect IFN-γ and IL-4 mRNA expression. CD4+CD25− T cells from B6 mice were stimulated with anti-CD3, anti-CD28, and the indicated cytokines in the presence or absence of Baicalin for 3 days. IFN-γ and IL-4 mRNA expression were analyzed by RT-PCR. These experiments were performed three times with similar results. (TIF) [file pone.0017164.s006.tif]
